# Evaluation of Metal Nanoparticles Using ICP‐OES in Hairs of Patients With Frontal Fibrosing Alopecia and Comparison With Control Group

**DOI:** 10.1111/jocd.70893

**Published:** 2026-06-11

**Authors:** Farnaz Ahmadpour, Yalda Nahidi, Ali Ahmadpour, Naser Tayyebi Meibodi, Maryam Emadzadeh

**Affiliations:** ^1^ Cutaneous Leishmaniasis Research Center, Faculty of Medicine Mashhad University of Medical Sciences Mashhad Iran; ^2^ Department of Chemical Engineering Ferdowsi University of Mashhad Mashhad Iran; ^3^ Department of Pathology Mashhad University of Medical Sciences Mashhad Iran; ^4^ Clinical Research Development Unit Mashhad University of Medical Sciences Mashhad Iran

**Keywords:** frontal fibrosing alopecia, haircare products, nanoparticle, skin care products, titanium

## Abstract

**Background:**

Frontal fibrosing alopecia (FFA) is an increasingly prevalent scarring alopecia potentially linked to environmental factors such as nanoparticles (NPs) in skincare and sunscreens.

**Objectives:**

This case–control study aimed to quantify titanium, zinc, aluminum, and iron concentrations in hair shafts of FFA patients versus controls using inductively coupled plasma‐optical emission spectrometry (ICP‐OES).

**Methods:**

Thirty newly diagnosed, untreated FFA patients and 30 age‐ and sex‐matched controls were recruited from a dermatology clinic (February 2020–August 2021). Hair samples (15 shafts, up to 10 cm) were collected from the frontal hairline postcleansing. Elemental concentrations were measured in parts per million (ppm) after acid dissolution. Usage histories of sunscreens, cosmetics, and haircare products were recorded via checklist. Data were analyzed using SPSS v26.0, with nonparametric tests for comparisons (*p* < 0.05 significant).

**Results:**

FFA patients showed significantly higher hair shaft concentrations of titanium (*p* < 0.001), aluminum (*p* < 0.001), zinc (*p* < 0.001), and iron (*p* < 0.001) compared to controls. The FFA group reported more frequent use of hair bleaching (*p* = 0.038), haircare products (*p* ≤ 0.002), sunscreens (*p* < 0.001), and cosmetics (*p* < 0.001), with no differences in skincare use (*p* = 0.209) or hair dyeing (*p* = 0.961). In controls, frequent sunscreen use correlated with higher titanium levels (*p* = 0.011). No correlations emerged between product use and elemental concentrations in FFA patients (*p* > 0.05), but aluminum levels correlated with alopecia severity (*p* = 0.011).

**Conclusions:**

These findings indicate a possible association between elevated hair shaft concentrations of certain metals (titanium, zinc, aluminum, and iron), potentially linked to the use of metal‐containing products and FFA. While this highlights potential clinical relevance for advising cautious reduction in exposure to titanium‐ and zinc‐containing products, further studies are needed to validate these associations using combined microscopy and dynamic follicle analyses.

## 1 Introduction

Frontal fibrosing alopecia (FFA) is characterized by a progressive, irreversible scarring alopecia that affects the frontal and temporal hairlines [1]. Although the disease mainly affects women between the ages of 55–70, it can also affect premenopausal women and men [2, 3]. FFA's histopathology is similar to that of lichen planopilaris, although its clinical manifestations differ from those of classic lichen planopilaris, and it is considered a distinct variant of lichen planopilaris [1, 4]. The disease is often associated with eyebrow and/or eyelash involvement, but body hair involvement is less common [5, 6]. This disease was originally reported by Kossard in 1994 [4] and has since become increasingly prevalent over the ensuing decades [7, 8]. However, epidemiological data on FFA are limited, and the triggers for FFA remain unclear. Several factors are implicated in the pathogenesis of FFA, including genetic predisposition [9, 10], hormonal factors [2, 11], and environmental factors [12]. It has been suggested that exogenous agents serving as a reservoir by penetrating the hair follicle may trigger immune responses [13]. Among these environmental factors, sunscreens and leave‐on cosmetic products are considered potential triggers for FFA [14]. The study by Aldoori et al. was the first to reveal higher consumption of facial skincare products, especially sunscreens, among patients with FFA, and it suggested a causative role for sunscreen chemicals in the pathogenesis of the disease [15], which was confirmed by subsequent studies showing similar findings [12, 16]. Moreover, in a notable case report, regrowth of the frontal hairline occurred after cessation of sunscreen use [17].

In some laboratory and animal studies, the relationship between common sunscreens (such as benzophenone) and endocrine changes has been investigated [18]. It has been suggested that certain molecules in facial products affect hormonal systems and are classified as endocrine disruptors [19].

Ever since nanoparticles (NPs), especially titanium dioxide NPs, were added as ultraviolet filters to sunscreens and skincare products, speculation has emerged about their potential role [14]. Studies have shown that some titanium dioxide microparticles and NPs in topical skincare products, such as sunscreens, can penetrate as far as the isthmus and elicit a lichenoid reaction [20]. Another hypothesis is that these NPs can suppress the anti‐inflammatory effect of sunlight, leading to a breakdown of the immune privilege of the hair follicle [21]. In 2018, Brunet‐Possenti et al. were the first to demonstrate the presence of titanium particles on the hair shaft of a patient with FFA [22]. The latest study, conducted by Lyakhovitsky et al., revealed a significant increase in particles containing titanium, chlorine, silicon, magnesium, and iron in the hair shafts of 20 patients with FFA using scanning electron microscopy (SEM) and energy‐dispersive X‐ray spectroscopy (EDX) [23]. Current data regarding the presence of NPs in the hair shafts of patients with FFA do not investigate the quantities of these particles, nor do they evaluate all potential particles used in cosmetics, skincare, and haircare products that may play a role in the pathogenesis of this increasingly prevalent disease. Moreover, there is a need to increase the sample size to achieve more definitive results.

In this study, we aimed to measure and compare the concentrations of NPs in the hair shafts of patients with FFA and controls using inductively coupled plasma‐optical emission spectrometry (ICP‐OES), which measures elemental concentrations in parts per million (ppm) to achieve more accurate results. To the best of our knowledge, this is the first study to evaluate NPs of titanium, zinc, aluminum, and iron, along with their concentrations, in the hair shafts of patients with FFA compared with controls using ICP‐OES.

## 2 Materials and Methods

### 2.1 Study Population

This case–control study was conducted at the Department of Dermatology, between February 2020 and August 2021. Patients with FFA were recruited from among those visiting the dermatology clinic at Imam Reza Hospital who agreed to participate. All included patients provided written informed consent before participating in the study, and the study adhered to the tenets of the Declaration of Helsinki. The study protocol was approved by the Regional Medical Ethics Committee at MUMS (IR.MUMS.MEDICAL.REC.1399.622).

The diagnosis of FFA was based on clinical examination by an experienced dermatologist specializing in hair diseases, which included the presence of typical scarring alopecia in the frontal hairline and additional features such as eyebrow and/or body hair loss and facial papules. This was confirmed by histopathological examination of a biopsy by a dermatopathologist. The control group consisted of individuals matched to the patients in terms of sex and age (±5 years). To avoid selection bias, they were randomly selected from hospital staff and other patients who visited the dermatology clinic for other reasons. Exclusion criteria included any known scalp or hair disorders. All control participants were selected and evaluated by a board‐certified dermatologist. A clinical examination was performed on each control individual prior to sampling to confirm the absence of any scalp or hair disorders.

In all participants, a history of use of cosmetics and skincare products (e.g., moisturizer, sunscreen, toner, cleanser, foundation), haircare products (e.g., shampoo, hair dye, hair dryer), and their frequencies of use was recorded using a checklist. These reported frequencies refer to participants' habits prior to the diagnosis of FFA, ensuring that the data reflect predisease exposure rather than postdiagnosis changes. The frequency of use of haircare products was categorized as follows: no use, sometimes (≤ twice weekly), or always (≥ twice weekly). For facial skincare products: no use or yes. For facial cosmetics: no use, sometimes (≤ twice monthly), usually (≥ once weekly), or always (every day).

Sunscreen use was categorized as: no use, sometimes (≤ once weekly), or always (≥ twice weekly). Hair treatments were categorized as follows: hairstyling or straightening (no, sometimes [once monthly], always [≥ once weekly]); dyeing (no, once a year, twice a year, every 2 months, every month, or twice monthly); hair bleaching (no or yes).

The extent of hair loss was evaluated in all FFA patients using the clinical severity scale adapted from Vañó‐Galván et al. (2019), which measures the area of cicatricial skin resulting from frontal and temporal hairline recession [2]. Five grades of severity were defined as follows: Grade 1 (< 1 cm), Grade 2 (1–2.99 cm), Grade 3 (3–4.99 cm), Grade 4 (5–6.99 cm), and Grade 5 (≥ 7 cm).

All participants were newly diagnosed patients who had not yet initiated any specific treatments for FFA, ensuring that hair samples reflected baseline conditions unaffected by targeted therapies. However, prior use of nonspecific treatments (e.g., general skincare or unrelated medications) could not be entirely excluded. Hair samples were collected from each patient immediately following diagnosis and prior to the commencement of any therapeutic regimen. This approach ensured that the elemental analyses reflected baseline concentrations unaffected by medical interventions.

### 2.2 Hair Sampling

From all participants, 15 hair shafts were clipped from the frontal hairline, starting from the scalp, to a length of up to 10 cm after cleaning the area with alcohol. Participants were asked to wash their faces before sampling to remove any cosmetic products or sunscreen. The samples were stored in plastic bags and transported to the Central Laboratory of University for measurement of the concentrations of titanium, zinc, aluminum, and iron.

### 2.3 Sample Preparation

After accurately weighing all samples, they were dissolved in a solution composed of nitric acid and hydrochloric acid. Hydrogen peroxide was added to accelerate the dissolution process, and sulfuric acid was added to release all titanium particles. All samples were analyzed using ICP‐OES (Spectro Arcos‐76 004 555, Germany), which measures elements, including heavy metals and alkaline elements, at concentrations in ppm. Thus, quantitative levels of titanium, zinc, aluminum, and iron were reported.

### 2.4 Statistical Analysis

A total of 30 patients with FFA and 30 matched controls were recruited. In the present study, SPSS version 26.0 (SPSS Inc./IBM Corp., Chicago, IL, USA) was used for statistical analyses. Qualitative data were presented as their frequencies and percentages. The Kolmogorov–Smirnov test was performed to evaluate the normality of the data distribution. For non‐normally distributed variables, the Mann–Whitney test was used to compare measurements between patients and controls, and the Kruskal–Wallis test was used for comparisons involving more than two independent groups. Fisher's exact test was applied to compare categorical variables between the groups. In all calculations, a *p*‐value < 0.05 was considered statistically significant.

## 3 Results

### 3.1 Demographic Data

In the current study, 30 patients with FFA and 30 controls matched for sex and age were enrolled. The mean age of the FFA group and the control group was 49.6 ± 10.6 years (range 20–72 years) and 47.7 ± 7.8 years (range 37–63 years), respectively. No significant difference was observed between the two groups regarding age distribution and marital status (*p* = 0.245). The two groups were evaluated for the frequency of use of skincare, haircare, and cosmetic products (Table 1). The frequency of hair dyeing was not significantly different between the two groups (*p* = 0.961), although the frequency of hair bleaching in the FFA group was significantly higher than in the control group (*p* = 0.038). Haircare products such as hair masks, oils, or conditioners (*p* = 0.001), hair dryers (*p* = 0.001), and straighteners (*p* = 0.002) were used significantly more frequently in the FFA group than in the control group. Moreover, patients with FFA used sunscreens (*p* < 0.001) and cosmetics (*p* < 0.001) significantly more frequently. However, the frequency of use of skincare products, including moisturizing creams, oils, antiwrinkle creams, and eye contour creams, was not significantly different between the two groups (*p* = 0.209).

**TABLE 1:** History of use of facial and hair cosmetics, sunscreens and hair care practices in patients with frontal fibrosing alopecia and controls obtained by questionnaire.

	FFA (*n* = 30) *n* (%)	Controls (*n* = 30) *n* (%)	*p* ^a^
Hair dyeing No Once yearly Twice a year Every 2 months Every month Twice monthly	2 4 (14.3) 8 (28.6) 8 (28.6) 4 (14.3) 4 (14.3)	2 4 (14.3) 8 (28.6) 9 (32.1) 5 (17.9) 2 (7.1)	0.961
Hair bleaching No Yes	11 (36.6) 19 (63.3)	20 (66.6) 10 (33.3)	0.038
Haircare products No use Sometimes (≤ twice weekly) Always (≥ twice weekly)	5 (16.7) 8 (26.7) 17 (56.7)	19 (63.3) 3 (10) 8 (26.7)	0.001
Hairdryer No use Sometimes (once monthly) Always (≥ once weekly)	4 (13.3) 15 (50) 11 (36.7)	17 (56.7) 10 (33.3) 3 (10)	0.001
Hair straightener No use Sometimes (once monthly) Always (≥ once weekly)	16 (53.3) 11 (36.7) 3 (10)	28 (93.3) 2 (6.7) 0 (0)	0.002
Sunscreen No use Sometimes (≤ once weekly) Always (≥ twice weekly)	0 (0) 6 (20) 24 (80)	12 (40) 7 (23.3) 11 (36.7)	< 0.001
Cosmetics No use Sometimes (≤ twice monthly) Usually (≥ once weekly) Always (every day)	7 (23.3) 14 (46.7) 2 (6.7) 7 (23.3)	22 (73.3) 8 (26.7) 0 (0) 0 (0)	< 0.001
Skincare products No use Yes	26 (86.7) 4 (13.3)	21 (70) 9 (30)	0.209

^a^ Statistically significant difference (*p* < 0.05).

### 3.2 Particle Analysis Using ICP‐OES

Data from the quantitative analysis of hair shafts are reported in Tables 2 and 3. Elements including titanium, zinc, aluminum, and iron were detected in both the FFA group and controls.

**TABLE 2:** Concentrations of particles in patients with frontal fibrosing alopecia and controls obtained using inductively coupled plasma mass spectrometry (ICP‐OES).

	FFA (*n* = 30)	Control (*n* = 30)	*p* ^b^
**Titanium (ppm)** ^**a**^ Mean ± SD Median (minimum–maximum)	461.1 ± 375.9 346 (31–1609)	5.4 ± 11.99 2.3 (0.5–66.1)	< 0.001
**Zinc (ppm)** Mean ± SD Median (minimum–maximum)	420.7 ± 244.2 355.5 (131–1285)	214.2 ± 194.5 162.5 (48–1149)	< 0.001
**Aluminum (ppm)** Mean ± SD Median (minimum–maximum)	3378.6 ± 4017.4 1812 (360–16 482)	79.3 ± 37.8 74.5 (24–156)	< 0.001
**Iron (ppm)** Mean ± SD Median (minimum–maximum)	2631 ± 1904.1 1947 (631–7200)	309.8 ± 178.7 259 (105–859)	< 0.001

Sample Name: sample 1		s		
Measure Date: 2019‐09‐02 15:10	Recalc. Date: 2019‐09‐02 15	State: Recalculated	Quality: Recalc	Total Dilution: 1.000000
Sample Identification				
Sample Name	Dilution	WEIGHT	Volume	
Sample 1	1.000000	0.0135	25	

^a^ Parts‐per‐million.

^b^ Statistically significant difference (*p* < 0.05).

**TABLE 3:** The table, generated by the ICP‐OES, presents the concentrations of various elements in a sample from the FFA patient group. Three concentration measurements were obtained for each element per sample, with the mean value for each element subsequently recorded. The elements analyzed include aluminum, calcium, iron, zinc, and titanium.

	Al.	Ca.	Fe.	Zn.	Ti.
Conc 1	318.589[ppm]	3393.54[ppm]	1268.38[ppm]	501.715[ppm]	91.150[ppm]
Conc 2	325.015[ppm]	3394.65[ppm]	1280.72[ppm]	530.683[ppm]	91.301[ppm]
Conc 3	337.322[ppm]	3474.13[ppm]	1311.50[ppm]	560.193[ppm]	94.339[ppm]
Conc MinRange	—	—	—	—	…
**Conc Mean**	**326.975[ppm]**	**3420.77[ppm]**	**1286.87[ppm]**	**530.864[ppm]**	**92.263[ppm]**
Conc MaxRange	—	—	—	—	…
**Reported**	**326.975[ppm]**	**3420.77[ppm]**	**1286.87[ppm]**	**530.864[ppm]**	**92.263[ppm]**

The concentrations of titanium, zinc, aluminum, and iron were significantly higher in the FFA group than in the control group (*p* < 0.001). The analysis demonstrated significantly higher mean concentrations of titanium in the FFA group compared with controls. The mean concentration of titanium was 85.4 times higher in patients with FFA than in controls. The concentrations of aluminum, iron, and zinc were 42.6, 8.5, and 1.96 times higher, respectively, in the FFA group than in controls.

No significant correlation was found between the frequency of hair dyeing or bleaching and the concentrations of the elements studied in either the FFA or control groups (*p* > 0.05).

There was no significant correlation between the frequency of sunscreen use and the concentrations of titanium, zinc, aluminum, and iron in the FFA group (*p* > 0.05).

Although in the control group, individuals who always used sunscreen had significantly higher titanium concentrations than those who used it sometimes or not at all (*p* = 0.011).

In addition, there was no significant correlation between the frequency of using haircare products and cosmetics and the concentration of titanium, zinc, aluminum, and iron in the FFA group (*p* > 0.05).

However, there was a significant correlation between the extent of hair loss and aluminum concentrations in patients with FFA. Specifically, patients with more severe alopecia had higher aluminum concentrations (*p* = 0.011).

## 4 Discussion

This study using ICP‐OES showed higher concentrations of titanium, zinc, aluminum, and iron in the hair shafts of patients with FFA, indicating a possible association or accumulation of these particles in the damaged hair shaft. The greatest differences were observed for titanium and aluminum, with concentrations 85.4 and 42.6 times higher, respectively, in the FFA group than in the control group. These findings indicate the association between these elements and the pathogenesis of FFA. Furthermore, we found a higher frequency of use of sunscreens, cosmetics, haircare products, hair bleaching, and hairstyling among patients with FFA compared to the control group.

There are conflicting studies on the relationship between FFA and the use of skincare products, haircare products, and sunscreens [14, 24, 25, 26, 27, 28]. According to a survey by Aldoori et al. [15], the use of sunscreen was significantly higher in patients with FFA than in the control group. Moreover, the use of moisturizers and foundations was higher in the patient group, although the difference was not significant [15]. Therefore, this study raised the possibility of a causal role for leave‐on skincare products containing sunscreen chemicals in the pathogenesis of FFA, a hypothesis supported by subsequent studies [29, 30]. In another study, the use of sunscreens and cosmetics was significantly higher in the FFA group than in controls; however, the use of moisturizers did not differ significantly between the two groups [17]. These findings align with our results, which indicate a higher frequency of use of sunscreens, cosmetics, haircare products, hair bleaching, and hairstyling among patients with FFA compared to the control group. Furthermore, it was noted that the application of sunscreens to the frontal hairline could play a role in the etiology of FFA [17]. In a case report, titanium NPs were found in the hair shafts of patients with FFA; these NPs are commonly used as components in sunscreens [22]. This aligns with the findings of the present study, which support an association between these NPs and FFA. Accordingly, we evaluated NPs containing titanium, zinc, aluminum, and iron in the hair shafts of patients with FFA and compared them with those in a control group.

In the current study, increased concentrations of titanium and zinc were found in the hair shafts of patients with FFA; these elements are used in sunscreens for their photocatalytic activity. Sunscreens contain various types of active UV filters, some of which can cause allergic contact dermatitis and photoallergic dermatitis [31]. Titanium dioxide is one of the most common components in cosmetics, added not only to sunscreens but also as a colorant and volumizer in other products [32, 33]. Under UV exposure, titanium dioxide exhibits significant photocatalytic activity, which can lead to oxidative reactions causing DNA damage and skin inflammation [34]. In addition, upon exposure to light, the genotoxic effects of NPs are enhanced through increased oxidative stress [35]. Zinc oxide, used in cosmetics and sunscreens, has similar photocatalytic properties. Due to its 40 nm particle size, it can penetrate into cells, affecting cellular surfaces [20]. Therefore, based on a study by Thompson et al., it was hypothesized that titanium dioxide in cosmetics may penetrate to the follicular isthmus and potentially contribute to a lichenoid reaction at the follicular bulge, which could be associated with the development of FFA [20]. In another study, the depth of penetration of titanium NPs was estimated to be 400 μm into the hair follicle [36]. The induction of contact sensitivity and granulomatous reactions by TiO_2_ indicates that it is a target allergen for T lymphocytes [37]. Another hypothesis is that sunscreens suppress the anti‐inflammatory and immunomodulatory effects of sunlight [21]. These hypotheses ultimately lead to the breakdown of the hair follicle's immune privilege and the initiation of an autoimmune process against the hair follicle.

Our results regarding titanium are consistent with a recent study by Lyakhovitsky et al., which reported titanium NPs to be 8.6 times higher in patients with FFA than in controls using SEM and EDX, independent of age and disease duration [23]. Moreover, they reported a higher incidence and degree of cuticle weathering in patients with FFA, which could be due to mechanical, chemical, and physical factors or genetic predisposition. This suggests that the hair shafts of patients with FFA may be more susceptible to mechanical damage, potentially making them more permeable to NPs [23]. In addition, excessive use of hair bleaching compounds, such as hydrogen peroxide and ammonium persulfate, can lead to scalp burns, irregular hair cuticle scales, and impaired hair strength [38]. This damage and the associated oxidative stress resulting from hair bleaching compounds [39] can potentially increase the penetration of topical compounds applied to the skin and scalp. These factors could explain our finding that the more frequent hair bleaching reported among patients with FFA is associated with higher absorption or retention of NPs in their hair, possibly due to structural damage. Moreover, mitochondrial dysfunction in fatty acid oxidation, increased antioxidant production, has been reported to be involved in the pathogenesis of FFA [40]. Therefore, we hypothesize that this process might be linked to the effects of titanium and zinc NPs on oxidative stress, though further evidence is needed.

Recent advancements in FFA research continue to explore the role of environmental exposures, particularly NPs in sunscreens and skincare products. A 2025 comprehensive review highlights the potential for titanium dioxide NPs to accumulate on hair shafts, which may be associated with disrupting hair follicle immune privilege through residue buildup from infrequent shampooing. Similarly, studies on cosmeceuticals suggest that zinc oxide and titanium‐based filters may contribute to inflammation, recommending allergen‐free alternatives for management. These findings, including a systematic review from 2024 that questions the causal link between sunscreens and FFA and attributes observed associations to secondary factors like actinic damage, underscore the need for precise quantification of elemental concentrations, as pursued in the current study using ICP‐OES.

Furthermore, emerging evidence from 2024 case–control studies indicates a higher prevalence of contact allergens, including those in titanium‐containing products, among FFA patients, with particle deposition on hair shafts potentially exacerbating scarring. Reviews on cosmetic dermatology approaches advocate avoiding NP‐laden skincare while noting methodological limitations in prior qualitative analyses like SEM/EDX. By quantifying titanium, zinc, aluminum, and iron in ppm via ICP‐OES, our research addresses these gaps, providing more definitive data on baseline exposures in newly diagnosed cases supporting an association with environmental factors in FFA etiology, though without establishing causation.

To achieve more accurate results, we increased the sample size and used ICP‐OES to analyze the hair shafts, quantifying elemental concentrations in ppm. To the best of our knowledge, these elements have not been previously quantified using ICP‐OES, making our study unique in this regard. All patients in the present study were newly diagnosed cases. This approach eliminated potential bias from topical treatments that could affect hair cuticles and distort the results.

In addition to NPs containing titanium and zinc, this study revealed significantly higher concentrations of aluminum and iron in the hair shafts of patients with FFA. Aluminum is present in cosmetics and healthcare products, including sunscreens, toothpaste, lipsticks, and antiperspirants, potentially leading to oral and dermal absorption [41]. Iron oxide is also used as a colorant and pigment in cosmetics, including foundations, hair dyes, and lipsticks [42]. Given their cytotoxic effects through increased free radical production in human stem cells [43], we hypothesize that these NPs in cosmetics could potentially be associated with a role similar to that of titanium in FFA, though without proving causation, as higher levels may reflect retention in damaged hair. The higher levels of iron detected in patients with FFA align with a previous study, although that study did not report elevated aluminum levels in patients with FFA compared to controls [23]. In addition, we found significantly higher aluminum concentrations associated with higher grades of alopecia in patients with FFA. This is a unique finding that should be addressed in further studies for validation.

The present study found no significant correlation between the frequency of hair dyeing, bleaching, sunscreen use, haircare products, and cosmetics and the concentrations of the elements studied in either the FFA or control groups. However, in the control group, individuals who always used sunscreen had significantly higher titanium concentrations than those who used it sometimes or not at all. These findings could result from higher absorption or stability of NPs in the hair shafts of patients with FFA due to structural damage or genetic predisposition, or alternatively, from greater particle retention in FFA‐affected hair, independent of the number of products used or the undetermined amounts applied by patients. Therefore, exploring this aspect further should be a goal of future research due to its novelty.

One limitation of the present study is recalling bias. Patients may not accurately remember using a particular product or the extent of its application. Furthermore, information about the exact ingredients of the products used and their amounts is impossible to obtain. This study examined the presence and concentrations of titanium particles and other elements in the entire hair shaft and, therefore, could not prove their entry into the hair follicle.

In summary, our results revealed higher concentrations of NPs containing titanium, zinc, aluminum, and iron in the hair shafts of patients with FFA compared to controls. These findings suggest an association between skincare, haircare products, and cosmetics containing these NPs and FFA, though they do not establish a causal role, as it is possible that FFA‐affected hair or scalp retains more particles. It may be reasonable to advise patients with FFA to consider reducing excessive use of these products, pending further studies.

The use of combined methods to study the morphology and structure of hair and the location of these elements in the hair shaft, using transmission electron microscopy (TEM), SEM, and ICP‐OES, can help achieve more comprehensive results. Dynamic studies on plucked hair follicles after the application of creams containing NPs, including measuring particles in different areas of the hair follicle and examining their exact locations, should be a focus of future research on this increasingly prevalent disorder.

## Author Contributions

The Corresponding author, Dr. Yalda Nahidi, takes full responsibility for this work and all of the authors contributed equally to this work.

## Funding

The authors appreciate the financial support of Mashhad University of Medical Sciences, Iran (Grant No. 981228) for this work.

## Ethics Statement

The study protocol was approved by the Regional Medical Ethics Committee at MUMS (IR.MUMS.MEDICAL.REC.1399.622) conducted according to the Declaration of Helsinki principles. All participants provided written informed consent before participating in this investigation.

## Conflicts of Interest

The authors declare no conflicts of interest.

## Data Availability

The data that support the findings of this study are available from the corresponding author upon reasonable request.

## References

[1] S. Kossard , M.‐S. Lee , and B. Wilkinson , “Postmenopausal Frontal Fibrosing Alopecia: A Frontal Variant of Lichen Planopilaris,” Journal of the American Academy of Dermatology 36, no. 1 (1997): 59–66. 8996262 10.1016/s0190-9622(97)70326-8

[2] S. Vañó‐Galván , A. M. Molina‐Ruiz , C. Serrano‐Falcón , et al., “Frontal Fibrosing Alopecia: A Multicenter Review of 355 Patients,” Journal of the American Academy of Dermatology 70, no. 4 (2014): 670–678. 24508293 10.1016/j.jaad.2013.12.003

[3] B. Ladizinski , A. Bazakas , M. A. Selim , et al., “Frontal Fibrosing Alopecia: A Retrospective Review of 19 Patients Seen at Duke University,” Journal of the American Academy of Dermatology 68, no. 5 (2013): 749–755. 23375454 10.1016/j.jaad.2012.09.043

[4] S. Kossard , “Postmenopausal Frontal Fibrosing Alopecia Scarring Alopecia in a Pattern Distribution,” Archives of Dermatology 130, no. 6 (1994): 770–774. 8002649

[5] D. Moreno‐Ramírez and M. F. Camacho , “Frontal Fibrosing Alopecia: A Survey in 16 Patients,” Journal of the European Academy of Dermatology and Venereology 19, no. 6 (2005): 700–705. 16268874 10.1111/j.1468-3083.2005.01291.x

[6] A. Samrao , A.‐L. Chew , and V. Price , “Frontal Fibrosing Alopecia: A Clinical Review of 36 Patients,” Brit J Dermatol 163, no. 6 (2010): 1296–1300. 20698851 10.1111/j.1365-2133.2010.09965.x

[7] A. MacDonald , C. Clark , and S. Holmes , “Frontal Fibrosing Alopecia: A Review of 60 Cases,” Journal of the American Academy of Dermatology 67, no. 5 (2012): 955–961. 22503342 10.1016/j.jaad.2011.12.038

[8] P. Mirmirani , A. Tosti , L. Goldberg , et al., “Frontal Fibrosing Alopecia: An Emerging Epidemic,” Skin Appendage Disorders 5, no. 2 (2019): 90–93. 30815440 10.1159/000489793 PMC6388564

[9] C. Tziotzios , D. A. Fenton , C. M. Stefanato , et al., “Familial Frontal Fibrosing Alopecia,” Journal of the American Academy of Dermatology 73, no. 1 (2015): e37. 26089074 10.1016/j.jaad.2015.01.057

[10] C. Tziotzios , C. Petridis , N. Dand , et al., “Genome‐Wide Association Study in Frontal Fibrosing Alopecia Identifies Four Susceptibility Loci Including HLA‐B*07:02,” Nature Communications 10, no. 1 (2019): 1150. 10.1038/s41467-019-09117-w PMC6408457 30850646

[11] G. C. Ranasinghe , M. P. Piliang , and W. F. Bergfeld , “Prevalence of Hormonal and Endocrine Dysfunction in Patients With Lichen Planopilaris (LPP): A Retrospective Data Analysis of 168 Patients,” Journal of the American Academy of Dermatology 76, no. 2 (2017): 314–320. 28088992 10.1016/j.jaad.2016.05.038

[12] O. M. Moreno‐Arrones , D. Saceda‐Corralo , A. R. Rodrigues‐Barata , et al., “Risk Factors Associated With Frontal Fibrosing Alopecia: A Multicentre Case–Control Study,” Clinical and Experimental Dermatology 44, no. 4 (2019): 404–410. 30259544 10.1111/ced.13785

[13] M. Iorizzo and A. Tosti , “Frontal Fibrosing Alopecia: An Update on Pathogenesis, Diagnosis, and Treatment,” American Journal of Clinical Dermatology 20, no. 3 (2019): 379–390. 30659454 10.1007/s40257-019-00424-y

[14] G. Robinson , A. McMichael , S. Q. Wang , et al., “Sunscreen and Frontal Fibrosing Alopecia: A Review,” Journal of the American Academy of Dermatology 82, no. 3 (2020): 723–728. 31654665 10.1016/j.jaad.2019.09.085

[15] N. Aldoori , K. Dobson , C. R. Holden , et al., “Frontal Fibrosing Alopecia: Possible Association With Leave‐On Facial Skin Care Products and Sunscreens; a Questionnaire Study,” Brit J Dermatol 175, no. 4 (2016): 762–767. 26987767 10.1111/bjd.14535

[16] W. Cranwell , “Sinclair R, Editors The Role of Sunscreen and Facial Skin Care Products in Frontal Fibrosing Alopecia,” Australasian Journal of Dermatology 58 (2017): 48–49.

[17] W. C. Cranwell and R. Sinclair , “Frontal Fibrosing Alopecia: Regrowth Following Cessation of Sunscreen on the Forehead,” Australasian Journal of Dermatology 60, no. 1 (2019): 60–61. 29896853 10.1111/ajd.12833

[18] J. Wang , L. Pan , S. Wu , et al., “Recent Advances on Endocrine Disrupting Effects of UV Filters,” Int J Env Res Pub he 13, no. 8 (2016): 782. 10.3390/ijerph13080782 PMC4997468 27527194

[19] R. H. Waring and R. M. Harris , “Endocrine Disrupters: A Human Risk?,” Molecular and Cellular Endocrinology 244, no. 1 (2005): 2–9. 16271281 10.1016/j.mce.2005.02.007

[20] C. T. Thompson , Z. Q. Chen , A. Kolivras , et al., “Identification of Titanium Dioxide on the Hair Shaft of Patients With and Without Frontal Fibrosing Alopecia: A Pilot Study of 20 Patients,” Brit J Dermatol 181, no. 1 (2019): 216–217. 30648256 10.1111/bjd.17639

[21] W. C. Cranwell and R. Sinclair , “Sunscreen and Facial Skincare Products in Frontal Fibrosing Alopecia: A Case–Control Study,” Brit J Dermatol 180, no. 4 (2019): 943–944. 30367472 10.1111/bjd.17354

[22] F. Brunet‐Possenti , L. Deschamps , H. Colboc , et al., “Detection of Titanium Nanoparticles in the Hair Shafts of a Patient With Frontal Fibrosing Alopecia,” Journal of the European Academy of Dermatology and Venereology 32, no. 12 (2018): e442‐e3. 29578625 10.1111/jdv.14967

[23] A. Lyakhovitsky , E. Kartvelishvily , T. Drousiotis , et al., “Hair Shaft Morphology, Elemental Composition, and Nanoparticles in Frontal Fibrosing Alopecia: A Case‐Control Study,” Acta Dermato‐Venereologica 101, no. 9 (2021): adv00541. 34396423 10.2340/00015555-3891 PMC9425563

[24] M. Landau , S. M. Perez , and A. Tosti , “Frontal Fibrosing Alopecia: A Comprehensive Guide for Cosmetic Dermatologists,” Dermatol. Ther. (Heidelb.) 15, no. 1 (2025): 15–29. 39607666 10.1007/s13555-024-01311-z PMC11785866

[25] K. K. Verma , B. Fenner , M. Pham , et al., “Common Contact Allergens Implicated in Frontal Fibrosing Alopecia Found in Over‐The‐Counter Hair Growth Serums and Solutions,” Int J Womens Dermatol 10, no. 2 (2024): e149. 38783993 10.1097/JW9.0000000000000149 PMC11111391

[26] S. Verma , A. Marak , and D. Paul , “Frontal Fibrosing Alopecia: A Comprehensive Review With Recent Updates,” Indian Journal of Dermatology 70, no. 2 (2025): 115. 10.4103/ijd.ijd_419_24 PMC11952711 40162350

[27] D. Carrion‐Alvarez , K. Robles‐Estrada , A. I. Trejo‐Castro , et al., “Frontal Fibrosing Alopecia: Is Sunscreen the Culprit? A Systematic Review,” Iranian Journal of Dermatology 27, no. 3 (2024): 181–186.

[28] M. L. Porriño‐Bustamante , T. Montero‐Vílchez , F. J. Pinedo‐Moraleda , et al., “Frontal Fibrosing Alopecia and Sunscreen Use: A Cross‐Sectional Study of Actinic Damage,” Acta Dermato‐Venereologica 102 (2022): adv00757. 35604235 10.2340/actadv.v102.306 PMC9609976

[29] P. M. Ramos , A. Anzai , B. Duque‐Estrada , et al., “Risk Factors for Frontal Fibrosing Alopecia: A Case‐Control Study in a Multiracial Population,” Journal of the American Academy of Dermatology 84, no. 3 (2021): 712–718. 32835739 10.1016/j.jaad.2020.08.076

[30] A. Debroy Kidambi , K. Dobson , S. Holmes , et al., “Frontal Fibrosing Alopecia in Men: An Association With Facial Moisturizers and Sunscreens,” Brit J Dermatol 177, no. 1 (2017): 260–261. 28112792 10.1111/bjd.15311

[31] A. R. Heurung , S. I. Raju , and E. M. Warshaw , “Adverse Reactions to Sunscreen Agents: Epidemiology, Responsible Irritants and Allergens, Clinical Characteristics, and Management,” Dermatitis 25, no. 6 (2014): 289–326. 25384223 10.1097/DER.0000000000000079

[32] T. G. Smijs and S. Pavel , “Titanium Dioxide and Zinc Oxide Nanoparticles in Sunscreens: Focus on Their Safety and Effectiveness,” Nanotechnology, Science and Applications 4 (2011): 95–112. 24198489 10.2147/NSA.S19419 PMC3781714

[33] A. Weir , P. Westerhoff , L. Fabricius , K. Hristovski , and N. von Goetz , “Titanium Dioxide Nanoparticles in Food and Personal Care Products,” Environmental Science & Technology 46, no. 4 (2012): 2242–2250. 22260395 10.1021/es204168d PMC3288463

[34] A. Fujishima and K. J. Honda , “Electrochemical Photolysis of Water at a Semiconductor Electrode,” Nature 238, no. 5358 (1972): 37–38. 12635268 10.1038/238037a0

[35] B. S. Ayse , “Toxicity of Metal and Metal Oxide Nanoparticles: A Review,” Environmental Chemistry Letters 18, no. 5 (2020): 1659‐83‐2020.

[36] J. Lekki , Z. Stachura , W. Dąbroś , et al., “On the Follicular Pathway of Percutaneous Uptake of Nanoparticles: Ion Microscopy and Autoradiography Studies,” Nuclear Instruments and Methods in Physics Research B 260, no. 1 (2007): 174–177.

[37] K. Müller and E. Valentine‐Thon , “Hypersensitivity to Titanium: Clinical and Laboratory Evidence,” Neuro Endocrinology Letters 27, no. Suppl 1 (2006): 31–35. 17261997

[38] M.‐S. Jeong , C.‐M. Lee , W.‐J. Jeong , et al., “Significant Damage of the Skin and Hair Following Hair Bleaching,” Journal of Dermatology 37, no. 10 (2010): 882–887. 20860738 10.1111/j.1346-8138.2010.00916.x

[39] A. J. Grosvenor , S. Deb‐Choudhury , P. G. Middlewood , et al., “The Physical and Chemical Disruption of Human Hair After Bleaching—Studies by Transmission Electron Microscopy and Redox Proteomics,” International Journal of Cosmetic Science 40, no. 6 (2018): 536–548. 30229956 10.1111/ics.12495

[40] M. L. Porriño‐Bustamante , M. A. Fernández‐Pugnaire , and S. Arias‐Santiago , “Frontal Fibrosing Alopecia: A Review,” Journal of Clinical Medicine 10, no. 9 (2021): 1805. 33919069 10.3390/jcm10091805 PMC8122646

[41] T. Tietz , A. Lenzner , A. E. Kolbaum , et al., “Aggregated Aluminium Exposure: Risk Assessment for the General Population,” Archives of Toxicology 93, no. 12 (2019): 3503–3521. 31659427 10.1007/s00204-019-02599-z

[42] A. B. G. Lansdown , “Iron: A Cosmetic Constituent but an Essential Nutrient for Healthy Skin,” International Journal of Cosmetic Science 23, no. 3 (2001): 129–137. 18498465 10.1046/j.1467-2494.2001.00082.x

[43] A. A. Alshatwi , P. V. Subbarayan , E. Ramesh , et al., “Aluminium Oxide Nanoparticles Induce Mitochondrial‐Mediated Oxidative Stress and Alter the Expression of Antioxidant Enzymes in Human Mesenchymal Stem Cells,” Food Additives and Contaminants: Part A 30, no. 1 (2013): 1–10. 10.1080/19440049.2012.729160 23046173

